# Rural urban differences in self-rated health among older adults: examining the role of marital status and living arrangements

**DOI:** 10.1186/s12889-022-14569-9

**Published:** 2022-11-25

**Authors:** Amiya Saha, Margubur Rahaman, Bittu Mandal, Sourav Biswas, Dipti Govil

**Affiliations:** 1 Department of Family & Generations, International Institute for Population Sciences, Mumbai, 400088 India; 2 Department of Migration & Urban Studies, International Institute for Population Sciences, Mumbai, 400088 India; 3 School of Humanities and Social Sciences, Indian Institute of Technology, Indore, 453552 India; 4 Department of Population & Development, International Institute for Population Sciences, Mumbai, 400088 India

**Keywords:** Self-rated health, Rural–urban difference, Marital status, Living arrangements, Elderly

## Abstract

**Background:**

The rural–urban gap in socioeconomic and morbidity status among older adults is prevalent in India. These disparities may impact the levels and factors of self-rated health (SRH). The objective of the study is to compare the levels and determinants of SRH between rural and urban areas by considering the moderating effects of marital status and living arrangements.

**Subjects and methods:**

The present study used data from the Longitudinal Ageing Study in India (LASI) wave 1 (2017–18). A total sample of 30,633 older adults aged 60 years and above were selected for the study. Descriptive statistics, bivariate chi-square test, the interaction effect of living arrangements and marital status, and logistic estimation were applied to accomplish the study objectives.

**Results:**

The prevalence of poor SRH was found 7% higher in rural areas compared to urban counterparts. A substantial rural–urban disparity in the patterns of poor SRH was also observed. The interaction effect of marital status and living arrangement on self-rated health suggested that older adults who were currently unmarried and living alone were 38% more likely to report poor SRH than those who were currently married and co-residing in rural India. In addition to marital status and living situation, other factors that significantly influenced SRH include age, socio-cultural background (educational attainment and religion), economic background (employment status), health status (ADLs, IADLs, multi-morbidities), and geographic background (region).

**Conclusion:**

The present study's findings demonstrated that, notwithstanding local variations, marital status and living circumstances significantly influenced SRH in India. In the present study, unmarried older people living alone were more susceptible to poor SRH in rural areas. The present study supports the importance of reinforcing the concepts of care and support for older individuals. There is a need for special policy attention to older individuals, particularly those unmarried and living alone. Although older individuals had difficulty performing ADLs and IADLs and had multi-morbidities, they reported poorer health. Therefore, offering them social support and top-notch medical assistance is crucial.

## Background

The aging population growth rate suggests that lower-middle-income countries (LMICs) are expanding faster than their developed counterparts [1]. By 2050, less-developed regions will be home to almost 80% of the world's aging population. The aging population in the Asia–Pacific region has been growing speedily and is expected to reach 1.3 billion people in 2050 [2]. Similarly, India has been experiencing an unprecedented aging population among the Asia–Pacific nations and is expected to reach 319 million by 2050, representing nearly 20% of the overall population [2].

Self-rated health (SRH) is a standard measure of overall health status recommended by the World Health Organization (WHO). The level of SRH is significantly affected by both the physical and mental health of the individuals [3]. Apart from the Psycho-physical health status, the SRH is also significantly determined by various sociodemographic and behavioral aspects. Furthermore, the SRH significantly varies with geography (such as regional variation and rural–urban gap) found in many previous studies [4]. The global health status scenario displayed that poor SRH is mainly prevalent in LMICs [5]. In particular, the SRH is found to be poor with the increasing age of the individuals and a more concerning public health issue among the geriatric population compared to younger counterparts. Among the LMICs, including India, have been experiencing population structural change (rapid growth of older population) due to increasing life expectancy and lowering the Total Fertility Rate (TFR) [6]. Therefore, the increasing trend of geriatric population-inclined research focuses on health issues, healthcare utilization, and associated program and policies for the geriatric population. India has already taken various strategies and policies to achieve healthy ageing, although most older people responded to poor SRH and are more significant in rural areas [7].

The studies on ageing health and geography suggested that multi-morbidities are higher among the geriatric population, particularly in urban India [8]. Similarly, risky health behaviors and environmental health hazards were also higher among urban geriatric populations than in rural counterparts [9, 10]. However, the prevalence of difficulties in Activities of Daily Living (ADL) and Instrumental Activities of Daily Living (IADLs) are both higher among rural adults compared to urban settings [11]. Apart from health vulnerabilities, the geriatric population in rural areas is more socio-economically vulnerable than their urban counterparts. In particular, the lower level of education, poverty, and poor standard of living are common social diseases among the older population in rural areas, which are negatively associated with SRH found in previous studies [12, 13]. Further, the persistent inequality in the availability, accessibility, and quality of geriatric healthcare infrastructure between rural and urban areas also favors rural–urban differences in the health status of the older population. Therefore, the present study hypothesized that the SRH would be varied with the place of residence (rural/urban) with different sociodemographic patterns in India.

Several prior studies in Korea [14], Ghana [15], and Finland [12] examined the rural–urban differentials in levels and predictors of self-rated health. The studies found significant variation in prevalence and predictors of SRH in accordance with the place of residence. A recent study has examined the rural–urban gap in health status among the older population in India using large-scale sample survey data and found significant variation in predictors of successful aging between rural and urban [16]. However, it hasn't been investigated how the association between marital status and living arrangements with SRH varies depending on the place of residence in India. Prior research from other nations suggests that place, demographic context, and culture all have an impact on the linkage between married status and living arrangements with SRH [13]. Therefore, to fill this research gap, the present study investigates the rural–urban differences in self-rated health among older people, focusing on the role of marital status and living arrangements.

Existing literature suggests that marital status and living arrangements are significant social determinants of self-rated health in later life in India and other countries [17]. Many previous studies found a significant association between marital status and health outcomes among older people in India and elsewhere [18]. The positive and negative association varies with the geriatric population's space, place, and background characteristics [19]. Most studies found health status of the currently married elderly is better than unmarried or never-married counterparts. For example, married people have reported less loneliness and health issues than their unmarried/ever-married counterparts [13]. Secondly, studies from developed and developing nations highlighted that physical health status is comparatively better among married older than unmarried [20]. Third, marital status is considered important social status in many societies, positively reflecting individuals' mental health and social well-being [21]. Similarly, older who are living alone were found to be more vulnerable regarding health and socioeconomic well-being in India and elsewhere [18]. Further, physical and subjective health status also differs with individuals' socioeconomic and psycho-physical health backgrounds.

Hughes and Waite (2002) examined whether the household composition and living arrangements associated with marital status could explain health disparities between married groups [22]. They believed that marriage might have a protective role on risky health behaviors (decrease in risk behaviors such as smoking, excessive drinking, and alcohol abuse) because of social inclusion and social regulations [23] and have the positive provision of social and psychological support, as well as instrumental assistance for chores such as household work [24]. For example, Joung et al. (1997) suggested that the unhealthy categories of the intermediary factors were more prevalent among unmarried groups than among married ones [25]. The most significant was the lack of social support among never-married men and the negative financial situations of divorced women.

Geriatric health is an emerging concern in public health in India. Therefore, numerous previous studies examined predictors of SRH without considering disaggregate analysis based on place of residence (rural vs. urban) and found marriage is positively associated with better health and higher life satisfaction [26, 27]. The association between marital status, living arrangement, and SRH varies with the country because marriage norms and family dynamics are diverse in population, culture, and geography [28]. Therefore, the present study is relevant to exploring the rural–urban difference in the association between marital status and SRH among the older population by focusing on the living arrangement in the Indian context [29].

## Materials and methods

### Data source

The present study utilized data from the Longitudinal Ageing Study in India (LASI) wave 1 (2017–18), a nationwide and state-representative survey of aging and health. The first wave of the LASI surveyed 72,250 samples of individuals aged 45 and above, covering all 35 Indian states and union territories [30]. The main objective of the LASI survey is to provide longitudinal valid, reliable data on the geriatric population’s socioeconomic and health status, program and policy coverage status, and others. To arrive at the final units of observation, the LASI used a multistage stratified area probability cluster sampling design. LASI used a three-stage sample design in rural areas, while in urban areas, they used a four-stage sample design. The national report of LASI, wave 1, 2017–18, India, contains detailed information on the sampling framework and sample size selection [30].

### Study sample

The present study used secondary data, i.e., LASI Wave 1, which includes a total sample of 72,250 people aged 45 and above and their spouses, regardless of age, with no missing values in age reporting. The participants were selected using a multistage stratified area probability cluster sampling design. The face-to-face interviews were used to interview the respondents in their households [30].

**Fig. 1:**
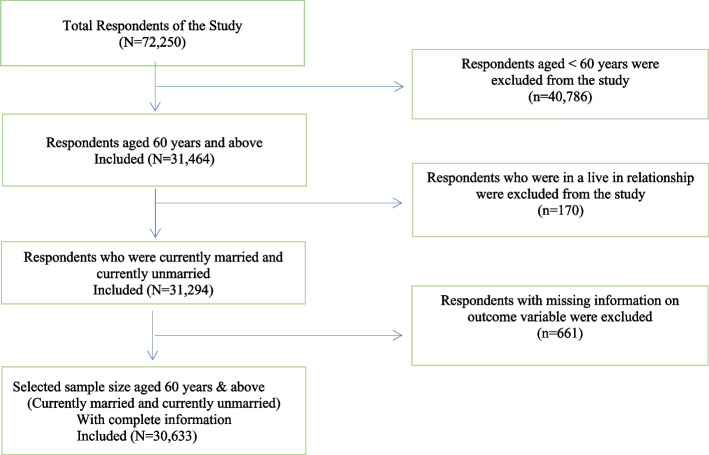
Graphical presentation of sample selection for the study

The participants were older individuals in our study, aged 60 and above, who were currently married, ever married, or unmarried. The final sample size of the study was 30,663 older individuals after excluding the respondents aged 59 years and below (*n* = 40,786), those who were in a live-in relationship (*n* = 170), and those who did not respond to self-rated health (*n* = 661). The details of the inclusion and exclusion criteria of the study sample are presented in Fig. 1. Since a live-in relationship is not treated as a married or unmarried status in India, we have removed it from the dataset considering Indian culture.

### Variable description

#### Outcome variable

##### Self-rated health

In the individual schedule, a question was asked to the respondents, *"Overall, how is your health in general?"* with responses of "Very good," "Good," "Fair," "Poor," and "Very poor." The outcome variable, i.e., self-rated health, is binary in nature in the present study. We considered fair, poor, and very poor as poor (coded as 1), whereas very good and good are considered good (coded as 0) [31].

#### Explanatory variables

##### Marital status and living arrangement

**Fig. 2:**
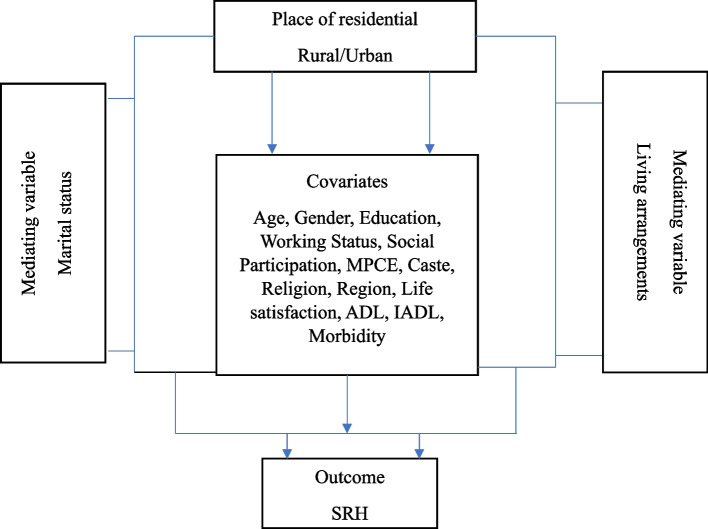
Preliminary conceptual model of rural–urban difference in SRH and its potential mediators of marital status and living arrangements

Contemporary evidence has categorized marital status into several categories: single, married, widowed, divorced, and separated [32]. However, our study aims at marriage and its role in the subjective health of an individual. It does not focus on other non-married categories despite knowing that the association across different categories of marriage may differ. Thus, our study has categorized marital status as binary classification with “1” those who responded married as “currently married” and all other categories as “2” those who responded widowed, never married, separated, divorced, and deserted as “currently unmarried.” The previous study suggests that living arrangement is a key determining factor of subjective health at later stage of life [33]. Therefore, the current study also included living arrangements as a key explanatory variable of SRH among the older population. Thus, the living arrangements of older adults have been categorized as binary classification with “1” those who responded living with spouse / or others, living with spouse and children, living with children and others, or living with others as “Co-residing” and “2” those who responded living alone has recorded as “Living alone” [34] (Fig. 2).

### Covariates

The analysis included and adjusted other sociodemographic, economic, and health-related characteristics (Fig. 2). Age was categorized as “60–69” years, “70–79” years, and “80 + ” years. Sex was categorized as male and female. Education was categorized as no schooling, up to the primary, up to secondary, and secondary & above. Working status was categorized as working and not working. Social participation was categorized as yes and no. Social participation was measured through the question, “Are you a member of any of the organizations, religious groups, clubs, or societies”? The response was categorized as yes and no. Life satisfaction was assessed among older adults using question a. In most ways, my life is close to ideal; b. The condition of my life is excellent; c. I am satisfied with my life; d. So far, I have got the important things I want in my life, e. If I could live my life again, I would change almost nothing. The responses were categorized as strongly disagree, somewhat disagree, slightly disagree, neither agree nor disagree, slightly agree, somewhat agree, and strongly agree. Using five statements, the life satisfaction scale was constructed as a ‘score of 5–20 as low satisfaction’, ‘score of 21–25 as medium satisfaction’, and ‘score of 26–35 as high satisfaction [35].

The six basic daily self-care activities that makeup activities of daily living include getting dressed, putting on chappals or shoes, walking across a room, bathing, eating, getting in or out of bed, and using the toilet, which includes getting up and down. Combining these six ADLs, a single variable was generated that was recorded as "no ADL" if the respondent had no difficulties performing any ADL and "ADL" if they had [35]. Additionally, IADLs included seven instrumental activity-related difficulties that were consistently performed. For example, preparing a hot meal (cooking and serving), shopping for groceries, making calls, taking medications, working in the garden or house, managing money by paying bills and keeping track of expenses, getting around or finding the address in a strange place were all taken into account when determining how well an individual could perform their instrumental activities of daily living (IADLs). IADLs were recorded as "no IADL" and "IADL," much like ADLs [35]. Morbidity status was categorized as 0, “no morbidity,” 1 as “single morbidity,” and 2 + as “multi-morbidity.” In the present study, we have measured financial condition based on the monthly per capita consumption expenditure (MPCE) computed and used as the summary measure of household expenditures. Sets of 11 and 29 questions on the expenditures on food and non-food items, respectively, were used to canvas the sample households. Food expenditure was collected based on a reference period of seven days, and non-food expenditure was collected based on reference periods of 30 days and 365 days. Food and non-food expenditures have been standardized to the 30-day reference period. Monthly per Capita Consumption Expenditure (MPCE) was coded as five quintiles, i.e., poorest, poorer, middle, richer, and richest [30]. Religion was coded as Hindu, Muslim, Christian, and others) [36]. Social group (Caste/Class) was coded as Scheduled Caste (SC), Scheduled Tribe (ST), Other Backward Class (OBC), and others. Caste is a well-known social stratification that leads to social prejudice afflicting lower castes (SCs, STs, and various sub-castes under OBCs) [36]. Caste-based discrimination is banned by legislation adopted under the Indian constitution. However, the practice of caste-based social exclusion is pervasive in India, which leads to caste-based inequalities in social and health well-being [37]. The place of residence was categorized as rural and urban. The region was coded as North, Northeast, Central, East, South, and West [30].

### Statistical approach

Descriptive statistics and bivariate analysis were used in this study to evaluate the prevalence of subjective health in the country based on socioeconomic status and other characteristics. The significance level of the bivariate correlation was determined using the Chi-square test. In addition, binary logistic regression analysis was used to examine the association between marital status, living arrangements, and subjective health in older people. The equation of the logistic regression is as follows:

$$\mathrm{Logit}(\mathrm Y)=\ln(\mathrm p/(1-\mathrm p))=\alpha+{\mathrm\beta}_1{\mathrm X}_1+{\mathrm\beta}_2{\mathrm X}_2+{\mathrm\beta}_3{\mathrm X}_3\dots...\beta_{\mathrm k}{\mathrm X}_{\mathrm k}+\;\mathrm\varepsilon\;$$

The regression coefficients in this example were β_1_, β_2_… … …β_k_, and they showed the relative effect of explanatory variables and sociodemographic and health behavioral factors on the dependent variable, with the coefficients changing depending on the context of the studies. The results from the adjusted odds ratio estimated the interaction effects of marital status and living arrangements on subjective health in older individuals in India. Interaction estimates have been adjusted for all other factors. The interaction effects (Marital status # Living arrangement) were used for the outcome variable and key explanatory variables, and the independent effects of marital status and living arrangement on subjective health were computed.

## Results

### Background characteristics of the study population

Table 1 provides the sample distribution of the study population. Overall, 24.08% (CI: 23.22–24.09) of olders in India reported poor SRH. In rural areas it was 25.09% (CI: 24.16 -26.04) and 21.53% (CI: 19.92 -23.23) in urban areas. Around 36.62% (CI: 35.59—37.65) of respondents were currently unmarried in rural areas compared to 40.97% (CI: 37.97—44.05) of older residing in urban areas. Nearly 5.75% (CI: 5.31–6.22) of older adults were living alone in India at the time of the survey. This figure was higher in rural areas at 6.40% (CI: 5.90—6.94) compared to urban areas at 4.16% (CI: 3.36—5.14). In rural areas, 77.03% (CI: 76.13—77.92) of older adults had no formal schooling, and 63.89% (CI: 62.85—64.92) of older adults reported they were not working, whereas, in urban areas, this figure was 46.24% (CI: 43.54—48.96) and 79.90% (CI: 78.07—81.61) respectively. Furthermore, 33.40% (CI: 32.48—34.53) of respondents in India reported low life satisfaction. It was a bit higher for the rural areas, 34.95% (CI: 33.96—35.97), than for urban areas, 28.40% (CI: 26.80—30.83). In rural areas, around 22.83% (CI: 21.94—23.74) and 47.46% (CI: 46.33—48.69), and in urban areas, 21.19% (CI: 19.33—23.17) and 21.19% (19.33—23.17) of older adults reported they had a problem in performing ADL and IADL activities, respectively. In rural areas, 18.85% (CI: 18.03—19.71) of the older adults reported multi-morbidity, whereas 35.97% (CI: 32.93—39.14) in urban areas. 21.34% (CI: 20.49—22.22) and 22.62% (CI: 20.82—24.52) of the elderly respondents living in rural and urban areas belonged to the poorest Monthly Per Capita Consumption Expenditure (MPCE) quintile, respectively.

**Table 1:** Sample distribution of older individuals (aged 60 years and above) by socio-demographic and economic backgrounds, India, LASI Wave 1 (2017–18)

**Background characteristics**	**India**	**Percent (95% CI)**	**Rural**	**Percent (95% CI)**	**Urban**	**Percent (95% CI** ^**a**^**)**
	**n**		**n**		**n**	
**Self-rated health**						
Good	23,264	75.95 [75.01 – 76.77]	16,258	74.91 [73.95 -75.84]	7,006	78.47 [76.77 – 80.07]
Poor	7,369	24.08 [23.22 – 24.90]	5,446	25.09 [24.16 -26.04]	1,922	21.53 [19.92 -23.23]
**Marital Status**						
Currently married	19,027	62.11 [60.93 – 63.28]	13,757	63.38 [62.34 – 64.40]	5,270	59.03 [55.95 – 62.03]
Currently unmarried	11,606	37.89 [36.72 – 39.07]	7,948	36.62 [35.59 – 37.65]	3,658	40.97 [37.97 – 44.05]
**Living Arrangement**						
Co-residing	28,871	94.25 [93.78 – 94.69]	20,315	93.60 [93.05 – 94.10]	8,557	95.84 [94.86 – 96.64]
Living alone	1,762	5.75 [5.31 – 6.22]	1,390	6.40 [5.90 – 6.94]	372	4.16 [3.36 – 5.14]
**Age group**						
60–69	1,822	59.42 [58.29—6.54]	12,871	59.30 [58.21 -60.38]	5,331	59.71 [56.86—62.50]
70–79	9,154	29.88 [28.81—3.99]	6,433	29.64 [28.62—30.68]	2,722	30.49 [27.79—33.32]
80 +	3,277	10.70 [10.3—11.4]	2,401	11.06 [10.36—11.81]	875	9.80 [8.36—11.47]
**Sex**						
Male	14,447	47.16 [46.62—48.27]	10,502	48.38 [47.30—49.47]	3,946	44.19 [41.59—46.83]
Female	16,186	52.84 [51.73—53.94]	11,203	51.61 [50.53—52.70]	4,983	55.81 [53.17—58.41]
**Education**						
No schooling	20,848	68.06 [66.87—69.23]	16,720	77.03 [76.13—77.92]	4,128	46.24 [43.54—48.96]
Up to primary	3,432	11.20 [10.63 -11.84]	2,167	9.98 [9.36—10.65]	1,265	14.17 [12.77—15.70]
Up to secondary	2,057	6.71 [5.87 -7.66]	1,174	5.41 [4.93—5.92]	883	9.89 [7.46 -12.99]
Secondary & above	4,296	14.20 [13.99—15.18]	1,644	7.58 [ 7.06—8.13]	2,652	29.70 [27.04—32.51]
**Working status**						
No	21,001	68.56 [67.60—69.52]	13,868	63.89 [62.85—64.92]	7,134	79.90 [78.07—81.61]
Yes	9,632	31.44 [30.50—32.43]	7,837	36.11 [35.08—37.15]	1,795	20.10 [18.39—21.93]
**Social participation**						
No	29,182	95.26 [94.95—95.64]	20,745	95.58 [95.20—95.92]	8,438	94.50 [93.61—95.28]
Yes	1,457	4.74 [4.40—5.99]	960	4.42 [4.08—4.80]	491	5.50 [4.72—6.39]
**Life satisfaction**						
Low	10,122	33.40 [32.48—34.53]	7,587	34.95 [33.96—35.97]	2,535	28.40 [26.80—30.83]
Medium	6,761	22.70 [21.27—22.92]	5,048	23.26 [22.37—24.17]	1,713	19.18 [17.53—20.95]
High	13,750	44.89 [43.75—46.33]	9,070	41.78 [40.71—42.87]	4,680	52.42 [49.66—55.17]
**Difficulty in ADL** ^**b**^						
No	23,640	77.17 [76.26—78.56]	16,603	76.50 [75.50—77.46]	7,036	78.81 [76.83—80.67]
Yes	6,993	22.83 [21.94—23.74]	5,101	23.50 [22.54—24.50]	1,892	21.19 [19.33—23.17]
**Difficulty in IADL** ^**c**^						
No	16,694	52.54 [51.39—53.67]	10,725	49.41 [48.33—50.49]	5,369	60.13 [57.01—63.18]
Yes	14,539	47.46 [46.33—48.69]	10,980	50.59 [49.51 -51.67]	3,559	39.87 [36.82—42.99]
**Morbidity status**						
0	14,372	46.92 [45.82—48.02]	11,495	52.96 [51.89—54.04]	2,876	32.22 [30.04—34.47]
1	8,957	29.24 [28.30—30.20]	6,117	28.18 [27.26—29.13]	2,840	31.81 [29.49—34.23]
2 +	7,305	23.84 [22.68—25.05]	4,092	18.85 [18.03—19.71]	3,212	35.97 [32.93—39.14]
**MPCE** ^**d**^ **quintile**						
Poorest	6,651	21.71 [20.99—22.54]	4,632	21.34 [20.49—22.22]	2,019	22.62 [20.82—24.52]
Poorer	6,641	21.68 [20.88—22.54]	4,822	22.21 [21.31—23.15]	1,819	20.38 [18.80—22.05]
Middle	6,352	20.74 [19.89—21.61]	4,596	21.17 [20.28—22.10]	1,756	19.67 [17.78—21.72]
Richer	5,932	19.36 [18.36—20.41]	4,128	19.02 [18.18—19.89]	1,804	20.20 [17.53—23.17]
Richest	5,057	16.51 [15.57—17.50]	3,527	16.25 [15.51 -17.02]	1,530	17.13 [14.54—20.08]
**Religion**						
Hindu	25,315	82.64 [81.94—83.32]	18,132	83.54 [82.74—84.31]	7,183	80.45 [78.94—81.88]
Muslim	3,306	10.79 [10.23—11.38]	2,105	9.70 [9.05—10.39]	1,200	13.44 [12.30—14.68]
Christian	883	2.88 [2.64—3.14]	653	3.01 [2.71—3.34]	230	2.58 [2.23—2.99]
Others	1,129	3.69 [3.39—3.99]	815	3.75 [3.42—4.12]	314	3.52 [2.99—4.14]
**Social group**						
Scheduled Caste	5,831	19.30 [18.28—19.82]	4,798	22.10 [21.20—23.03]	1,033	11.57[10.37—12.90]
Scheduled Tribe	2,476	8.80 [7.61—8.57]	2,200	10.14 [9.54—10.77]	276	3.09 [2.55—3.74]
Other backward Class	13,780	44.99 [43.83—46.15]	9,663	44.52 [43.44—45.61]	4,117	46.11 [43.19—49.06]
Others	8,546	27.90 [27.43—28.78]	5,044	23.24 [22.39—24.11]	3,502	39.23 [36.86—41.64]
**Region**						
North	2,387	7.79 [7.45—8.12]	1,614	7.44 [7.08—7.80]	773	8.66 [7.98—9.41]
North East	916	2.99 [2.83—3.16]	727	3.35 [3.16—3.55]	189	2.11 [1.85—2.41]
East	7,296	23.82 [22.98 -24.69]	5,966	27.49 [26.49 -28.50]	1,330	14.90 [13.68—16.12]
Central	6,419	20.95 [20.13—21.81]	5,122	23.60 [22.60—24.63]	1,297	14.52 [13.28—15.86]
West	6,866	22.42 [21.60—23.25]	4,271	19.68 [18.83—20.56]	2,595	29.07 [27.06—31.17]
South	6,749	22.3 [20.79—23.33]	4,005	18.45 [17.70—19.23]	2,744	30.73 [27.32—34.36]
Total sample	30,633	-	21,407	-	8,928	-

^a^*CI* Confidence Interval, ^b^*ADL* Activities of Daily Living, ^c^*IADL* Instrumental Activities of Daily Living, ^d^*MPCE* Monthly per Capita Consumption Expenditure

### Rural–urban differentials in the prevalence of poor SRH among individuals by marital status and living arrangement

**Fig. 3:**
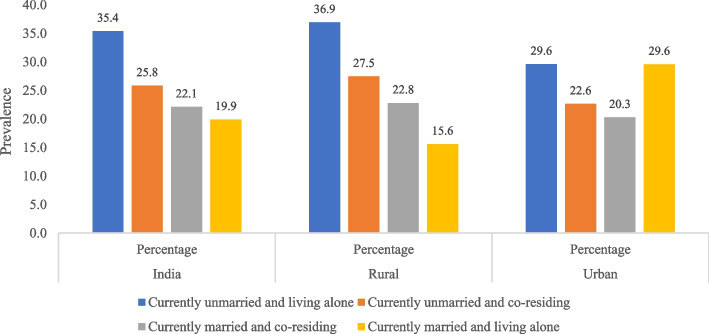
Prevalence of older adults reported poor health by marital status and living arrangement, India 2017–18

The prevalence of poor SRH was higher among currently unmarried older adults (27.27; 95% CI: 25.73—28.86]) than their currently married counterparts (22.10: 95% CI: 21.13—23.09) in India, irrespective of place of residence (Table 2). However, the difference in the prevalence of poor SRH between currently unmarried and married groups was found to be higher in rural (6.29%) compared to urban India (3.05%). In terms of living arrangements, the older adults living alone experienced higher poor SRH (35.10; 95% CI: 31.53—38.85) than their co-residing counterparts (23.38; 95% CI: 22.53—24.25) in India, especially in rural settings. The interaction result of marital and living arrangement status shows the prevalence of poor SRH was significantly high among the older adults who reported being currently unmarried and living alone than their counterparts in India, particularly in rural areas (Fig. 3).

**Table 2:** Prevalence of poor SRH by marital status and living arrangement among older individuals (aged 60 years and above), India, LASI Wave 1 (2017–18)

**Key explanatory variables**	**India**	**Rural**	**Urban**
	**Percent (95% CI)**	**Percent (95% CI)**	**Percent (95% CI)**
**Marital Status**	*p* < .001	*p* < .001	*p* < .001
Currently married	22.10 [21.13—23.09]	22.79 [21.67—23.95]	20.28 [18.47—22.23]
Currently unmarried	27.27 [25.73—28.86]	29.08 [27.48—30.74]	23.33 [20.35—26.60]
Difference	5.17	6.29	3.05
**Living Arrangement**	*p* < .001	*p* < .001	*p* < .001
Co-residing	23.38 [22.53—24.25]	24.31 [23.36—25.28]	21.18 [19.54—22.92]
Living alone	35.10 [31.53—38.85]	36.57 [32.63—40.70]	29.60 [22.32—38.09]
Difference	11.72	12.26	8.42

*P-*value based on Pearson Chi-square (Χ^2^) tests

### Rural–urban differentials in prevalence of poor SRH among older individuals by background characteristics

The prevalence of poor SRH increased with age in rural and urban India. Similarly, it was slightly higher among females compared to males in India, and the gap was more comprehensive in rural India. The prevalence was also observed to be substantially higher among individuals who did not have a formal education and was unemployed, had ADLs and IADLs, and had higher levels of morbidity, irrespective of place of residence (Table 3). For instance, it was 7% higher among unemployed older people in rural (30.32; 95% CI: 29.09–31.58) than in urban (23.55; 95% CI: 21.57–25.65) settings. Those who struggled with ADLs reported poor SRH nearly twice as compared to their peers, particularly in rural India (42.18; 95% CI: 39.82–44.59). Similarly, Christian community members reported higher poorer SRH than their counterparts. The prevalence was found to be comparatively higher in the southern region than in other areas, irrespective of whether rural or urban.

**Table 3:** Prevalence of poor SRH among older individuals (aged 60 years and above) by other background characteristics, India, LASI Wave 1 (2017–18)

	**India**	**Rural**	**Urban**
	**Percent (95% CI)**	**Percent (95% CI)**	**Percent (95% CI)**
	**24.08 [23.22 – 24.90]**	**25.09 [24.16 -26.04]**	**21.53 [19.92 -23.23]**
**Age**	*p* < .001	*p* < .001	*p* < .001
60–69	20.22 [19.28—21.19]	21.12 [20.24—22.24]	18.03 [16.32—19.90]
70–79	27.32 [25.59—29.16]	27.99 [26.13—29.94]	25.72 [22.13—29.67]
80 +	36.25 [33.31—39.30]	38.61 [35.33—41.99]	29.79 [24.26—35.95]
**Sex**	*p* < .001	*p* < .001	*p* < .001
Male	22.04 [20.94—23.18]	22.96 [21.67—24.30]	19.59 [17.53—21.83]
Female	25.85 [24.63—27.11]	27.09 [25.77—28.45]	23.07 [20.65—25.67]
**Education level**	*p* < .001	*p* < .001	*p* < .001
No schooling	26.23 [25.25—27.23]	26.40 [25.31—27.52]	25.51 [23.34—27.80]
Up to primary	23.64 [21.23—26.24]	22.29 [19.77—25.03]	25.95 [21.26—31.27]
Up to secondary	20.47 [17.04—24.39]	24.14 [20.37—28.35]	15.60 [10.87—21.88]
Secondary & above	15.56 [13.84—17.45]	16.13 [13.73—18.86]	15.21 [12.92—17.80]
**Working status**	*p* < .001	*p* < .001	*p* < .001
No	28.02 [26.92—29.14]	30.32 [29.09—31.58]	23.55 [21.57—25.65]
Yes	15.40 [14.26—16.62]	15.84 [14.53—17.24]	13.50 [11.45—15.87]
**Social participation**	*p* < .001	*p* < .001	*p* < .001
No	24.32 [23.46—25.20]	25.36 [24.40—26.35]	21.76 [20.08—23.55]
Yes	18.67 [16.14—21.49]	19.24 [16.24—22.66]	17.54 [13.27—22.82]
**Life satisfaction**	*p* < .001	*p* < .001	*p* < .001
Low	31.76 [30.24—33.32]	31.84 [30.25—33.46]	31.55 [27.83—35.51]
Medium	24.39 [22.84—26.01]	24.64 [22.83—26.55]	23.64 [20.74—26.81]
High	18.21 [17.06—19.43]	19.70 [18.27—21.21]	15.33 [13.57—17.27]
**Difficulty in ADL** ^**a**^	*p* < .001	*p* < .001	*p* < .001
No	19.14 [18.29—20.01]	19.84 [18.90—20.82]	17.47 [15.80—19.28]
Yes	40.68 [38.63—42.79]	42.18 [39.82—44.59]	36.63 [32.75—40.68]
**Difficulty in IADL** ^**b**^	*p* < .001	*p* < .001	*p* < .001
No	16.57 [15.63—17.55]	17.18 [16.06—18.35]	15.36 [13.70—17.18]
Yes	32.34 [30.86—33.85]	32.82 [31.38—34.30]	30.84 [27.01—34.96]
**Morbidity status**	*p* < .001	*p* < .001	*p* < .001
0	15.93 [14.92—17.00]	16.74 [15.57—17.97]	12.71 [10.86—14.82]
1	25.75 [24.34—27.21]	29.17 [27.47—30.92]	18.39 [16.24—20.76]
2 +	37.96 [35.41—40.57]	42.46 [40.05—44.91]	32.21 [27.94—36.81]
**MPCE** ^**c**^ **quintile**	*p* < .001	*p* < .005	*p* < .001
Poorest	25.98 [24.34—27.70]	26.55 [24.58—28.60]	24.69 [21.77—27.88]
Poorer	23.98 [22.37—25.62]	23.92 [21.98—25.98]	24.12 [21.39—27.07]
Middle	21.88 [20.18—23.68]	23.61 [21.55—25.81]	17.35 [14.77—20.27]
Richer	24.03 [22.07—26.10]	25.22 [23.18—27.39]	21.29 [17.38—25.81]
Richest	24.38 [22.09—26.83]	26.56 [24.32—28.93]	19.37 [14.65—25.16]
**Religion**	*p* < .001	*p* < .005	*p* < .001
Hindu	23.36 [22.43—24.31]	24.38 [23.36—25.43]	20.78 [18.92—22.77]
Muslim	26.77 [24.61—29.06]	28.16 [25.29—31.21]	24.35 [21.21—27.79]
Christian	36.36 [32.00—40.95]	34.72 [29.35—40.51]	40.99 [34.32—48.02]
Others	22.12 [18.71—25.95]	25.36 [21.11—30.14]	13.71 [9.40—19.56]
**Social group**	*p* < .001	*p* < .001	*p* < .001
Scheduled Caste	27.49 [25.62—29.45]	28.21 [26.10—30.41]	24.18 [20.45—28.34]
Scheduled Tribe	18.32 [16.34—20.48]	18.29 [16.21—20.58]	18.50 [12.84—25.90]
Other Backward Class	25.08 [23.67—26.54]	26.16 [24.68—27.69]	22.56 [19.62—25.78]
Others	21.72 [20.47—23.01]	23.05 [21.41—24.79]	19.79 [17.95—21.76]
**Region**	*p* < .001	*p* < .001	*p* < .001
North	22.88 [21.27—24.57]	23.99 [22.02—26.07]	20.57 [17.86—23.56]
North East	20.24 [18.14—22.53]	20.84 [18.49—23.41]	17.95 [13.62—23.29]
East	25.88 [24.27—27.56]	25.20 [23.40—27.09]	28.93 [25.53—32.59]
Central	23.18 [21.36—25.11]	24.04 [21.89—26.33]	19.81 [16.95—23.02]
West	16.83 [15.43—18.31]	19.24 [17.39—21.23]	12.86 [10.94—15.07]
South	31.19 [28.67—33.83]	33.74 [31.58—35.97]	27.47 [22.76 -32.75]

*CI* Confidence Interval, ^a^ *ADL* Activities of Daily Living, ^b^ *IADL* Instrumental Activities of Daily Living, ^c^ *MPCE* Monthly per Capita Consumption Expenditure

*P*-value based on Pearson Chi-square (Χ^2^) tests

### Rural–urban differentials in the association between marital and living status and SRH among older individuals

Marital status significantly predicted SRH in India (Table 4). A similar result was also observed in urban India, however insignificant in rural settings. Older unmarried individuals were 12% more likely (AOR: 1.12; 95% CI: 1.03–1.19) to report poor SRH than married women in urban India. Living arrangement was also significantly associated with SRH in India, particularly in rural settings. In rural India, older individuals living alone were 41% more likely (AOR: 1.41; 95% CI: 1.14–1.74) to experience poor SRH than their co-residing counterparts. The interaction effect of marital status and living arrangements on self-rated health was significant in India, particularly in rural settings. The study observed that older adults who were currently unmarried and living alone were 38% [AOR: 1.38; 95% CI: 1.11—1.71) more likely to experience poor health than those who were currently married and co-residing in rural settings.

**Table 4:** Unadjusted and adjusted odds ratios of poor SRH by selected key explanatory variables and other covariates and other covariates among older individuals (aged 60 years and above), India, LASI Wave 1 (2017–18)

**Key explanatory variables**	**UOR (95% CI)**	**India**	**UOR (95% CI)**	**Rural**	**UOR (95% CI)**	**Urban**
		**AOR (95% CI)**		**AOR (95% CI)**		**AOR (95% CI)**
**Marital Status**						
Currently married	Ref	Ref	Ref	Ref	Ref	Ref
Currently unmarried	1.32*** [1.20—1.46]	1.19*** [1.07—1.31]	1.39*** [1.25 -1.54]	1.02 [0.89—1.71]	1.20 [0.97—1.48]	1.12** [1.03—1.19]
**Living Arrangement**						
Co-residing with family	Ref	Ref	Ref	Ref	Ref	Ref
Living alone	1.77***[1.50—2.10]	1.48*** [1.20—1.83]	1.80*** [1.50—2.15]	1.41*** [1.14—1.74]	1.56* [1.06—2.32]	1.39 [0.83—2.32]
**Marital status **^a^** living arrangement**						
Currently Married ^a^ co-residing	Ref	Ref	Ref	Ref	Ref	Ref
Currently Married ^a^ Living alone	0.88 [0.37—2.06]	0.77 [0.32—1.88]	0.63 [0.22—1.80]	0.50 [0.17—1.47]	1.65 [0.37—7.42]	1.76 [0.38—8.12]
Currently Unmarried ^a^ Co-residing	1.23*** [1.11—1.36]	0.89 [0.78—1.02]	1.28*** [1.15—1.43]	0.96 [0.83—1.10]	1.15 [0.92—1.44]	0.79 [0.61—1.02]
Currently Unmarried ^a^ Living alone	1.93*** [1.62—2.29]	1.34 *** [1.08—1.65]	1.98*** [1.64—2.39]	1.38*** [1.11—1.71]	1.65** [1.10—2.48]	1.09 [0.65—1.82]
**Covariates**						
**Age**						
60–69		Ref		Ref		Ref
70–79		1.19*** [1.06—1.35]		1.17*** [1.03—1.33]		1.24 [0.97—1.59]
80 +		1.53*** [1.29—1.82]		1.55*** [1.29—1.88]		1.52*** [1.09—2.11]
**Sex**						
Male		Ref		Ref		Ref
Female		0.89 [0.79—1.00]		0.95 [0.84—1.08]		0.85 [0.67—1.09]
**Education level**						
No schooling		1.80***[1.44—2.23]		1.50*** [1.20—1.89]		1.59*** [1.16—2.20]
Up to primary		1.66*** [1.31—2.11]		1.33** [1.03—1.72]		1.87*** [1.28—2.74]
Up to secondary		1.43** [1.00—2.03]		1.69*** [1.26—2.25]		0.94 [0.55—1.61]
Secondary & above		Ref		Ref		Ref
**Working status**						
No		1.50*** [1.32—1.69]		1.58*** [1.38 -1.81]		1.52*** [1.17 – 1.97]
Yes		Ref		Ref		Ref
**Social participation**						
No		1.13 [0.93—1.38]		1.25** [1.00—1.57]		1.00 [0.68—1.49]
Yes		Ref		Ref		Ref
**Life satisfaction**						
Low		1.80*** [1.59—2.04]		1.69*** [1.48—1.93]		2.07*** [1.63—2.63]
Medium		1.35*** [1.18—1.54]		1.25*** [1.08—1.46]		1.53*** [1.19—1.96]
High		Ref		Ref		Ref
**Difficulty in ADL** ^**b**^						
No		Ref		Ref		Ref
Yes		1.98*** [1.75—2.25]		2.05*** [1.79—2.33]		1.84*** [1.42—2.38]
**Difficulty in IADL** ^**c**^						
No		Ref		Ref		Ref
Yes		1.40*** [1.25—1.58]		1.38*** [1.22—1.55]		1.43*** [1.12—1.83]
**Morbidity status**						
0		Ref		Ref		Ref
1		1.78*** [1.58—2.00]		1.91*** [1.69—2.16]		1.44*** [1.09—1.89]
2 +		3.12*** [2.74—3.56]		3.06*** [2.64—3.55]		3.44*** [2.68—4.422]
**MPCE** ^**d**^ **quintile**						
Poorest		1.06 [0.89—1.26]		1.01 [0.85 -1.21]		1.54** [1.04 -2.27]
Poorer		0.99 [0.83—1.17]		0.90 [0.75—1.09]		1.57** [1.09—2.28]
Middle		0.88 [0.74—1.05]		0.87 [0.73—1.04]		1.06 [0.71—1.58]
Richer		0.98 [0.81—1.20]		0.99 [0.83—1.17]		1.18 [0.76—1.83]
Richest		Ref		Ref		Ref
**Religion**						
Hindu		Ref		Ref		Ref
Muslim		1.13* [0.99—1.31]		1.24** [1.03—1.48]		1.16 [0.91—1.47]
Christian		1.85*** [1.46—2.35]		1.57*** [1.18—2.09]		2.70*** [1.86—3.92]
Others		0.95 [0.76—1.20]		1.02 [0.78—1.35]		0.78 [0.50—1.22]
**Social group**						
Scheduled Caste		1.29*** [1.11—1.48]		1.31*** [1.12—1.54]		1.26 [0.93—1.70]
Scheduled Tribe		0.95 [0.79—1.15]		0.96 [0.78—1.17]		1.22 [0.74—2.02]
Other backward Class		1.11 [0.98—1.26]		1.17** [1.02—1.35]		1.02 [0.81—1.29]
Others		Ref		Ref		Ref
**Region**						
North		0.98 [0,81—1.19]		0.93 [0.75—1.16]		1.15 [0.76—1.73]
North East		Ref		Ref		Ref
East		1.10 [0.92—1.32]		0.99 [0.81—1.22]		1.67*** [1.11—2.52]
Central		1.21 [0.99—1.46]		1.19 [0.96—1.49]		1.21 [0.79—1.85]
West		0.69*** [0.57—0.83]		0.76** [0.61—0.95]		0.62** [0.41—0.94]
South		1.30*** [1.07—1.58]		1.43*** [1.16—1.76]		1.37 [0.90—2.09]

*Ref* Reference, *UOR* Unadjusted Odds Ratio, *AOR* Adjusted Odds Ratio, *CI* Confidence Interval, *ADL* Activities of Daily Living, *IADL* Instrumental Activities of Daily Living, *MPCE* Monthly per Capita Consumption Expenditure

^a^ Interaction

^***^ *p* ≤ 0.001, ** *p* < 0.005 and * *p* ≤ 0.05

### Other determinants of SRH among older individuals by place of residence

The adjusted estimation of poor self-rated health with different socioeconomic and demographic characteristics revealed that increasing age was significantly and positively associated with poor health among older adults living in urban and rural areas. Older adults from rural and urban areas who were not working reported 58% [AOR: 1.58; CI: 1.38–1.81] and 52% [AOR: 1.52; CI: 1.17–1.97] significantly poor health than those who were working. Older adults from rural and urban areas who reported low life satisfaction were 1.69 times [AOR: 1.69; CI: 1.48–1.93] and 2.07 times [AOR: 2.07; CI: 1.63–2.63] more likely to have poor health in comparison to those who reported high life satisfaction respectively. The likelihood of poor health was 2.05 [AOR: 1.79—2.33] and 1.84 [AOR: 1.42—2.38] times higher among rural and urban community-dwelling older adults who had difficulty with ADL activities than those who did not have any difficulty with ADL activities. Similarly, in rural and urban areas, older adults with difficulty in IADL 38% and 43% were significantly more likely to report poor health than older adults who did not have difficulty with IADL activities. Having one or multiple morbid conditions had a strong positive association with the likelihood of poorer health in both rural and urban areas. Regarding the northeast region, respondents from the southern region reported 30% more likely poor SRH. Similarly, It was found to be 43% (AOR: 1.43; 95% CI: 1.16—1.76) more likely in the rural settings in the southern region.

## Discussion

The present study examined the differentials in prevalence and determinants of poor SRH among older individuals in India with a particular focus on marital status and living arrangements. According to the study findings, there was slight difference in self-rated health status between rural and urban areas in India. The result is similar to many previous studies in India [31]. However, the rural–urban difference in the prevalence of poor SRH was visible based on sex, marital status, and living arrangement. The prevalence of poor SRH was higher among females in rural areas than their urban counterparts. The most plausible explanation is that women's autonomy is lower among females in rural areas and is negatively associated with healthcare decision-making, utilization, and well-being [31]. The patient choice-centric availability of healthcare providers is also limited in rural settings, which negatively affects women's utilization of healthcare services and quality of care [9].

Regarding marital status, the likelihood of poor SRH was significantly higher among the currently unmarried people than their counterparts in India. The result is similar to many previous studies in India and elsewhere [18, 38]. The theory of marriage protection and selection explains the linkage between marital status and health outcomes, which supports our study findings. According to research on marriage's protective effects, marriage helps people maintain good health by shielding them from physical and emotional strain as well as harmful health behaviors [20, 38, 39]. Additionally, marriage deepens social bonds and social support, which enhances health.

On the other hand, the marriage selection theory argues that individuals who are healthier more likely to marry [40]. Therefore, healthier married individuals have a lower chance of both physical and psychological morbidities, which positively affect self-rated health status. In line with the above study, marital status was found to be a significant determinant of SRH among the older population in India, particularly in urban settings. In contrast, the association was found to be insignificant in rural settings. Therefore, the finding suggests a need for further study to explore why the result is insignificant in rural settings.

Existing studies of the moderating role of marriage and living arrangements on self-rated health may not differ among older adults conducted in modern western countries, where gender norms and social dynamics are different from India [41]. In rural areas, people are more socially well-connected; thus, the effect of marriage on health may not be significant, but in urban areas where most of the time, older reside in a nuclear family, and thus, marital status may affect their health status [42]. In line with earlier studies in India [43] and elsewhere, the current study discovered that poor SRH was more common among older adults living alone in India. However, SRH was significantly subjective by living arrangement status, particularly in rural areas, although insignificant in urban settings. In urban India, the level of family ties and support is low compared to rural counterparts, and older individuals are more likely to be independent in making healthcare and others decisions. When individuals are socio-economically independent and do not depend on family support in the later stages of life, the role of living arrangements becomes insignificant [26].

In our study, the adjusted interaction effect of marital status and living arrangement showed that the currently unmarried individuals living alone had a significantly higher experience of poor SRH than their currently married and co-residing counterparts in India, particularly in rural settings. It has been found that unmarried and living-alone individuals in rural settings are less likely to access quality healthcare facilities than urban counterparts [9]. It is challenging for people to access healthcare from rural settings, especially when single and living alone. One probable explanation is that individuals at later stages become more reliant on their family members in rural settings, whether financially, physically, or emotionally. In this case, if they are unable to receive any support from family members, their health will deteriorate [44].

Older individuals’ age, level of education, and working status are all predictors of SRH in India, irrespective of place of residence. The result is similar to many previous studies in India and elsewhere [43, 45]. The oldest-older (80 years and above), lower educated, and unemployed individuals are more likely to experience poor health than their counterparts. With increasing age, the risk of both communicable and non-communicable diseases is high, negatively affecting SRH [16]. Similarly, the lower educated and unemployed are more vulnerable to socio-economic distress, positively associated with poor SRH [45].

Similar to previous studies [46], social participation is a protective factor against poor SRH among older individuals, particularly in rural settings. Older psycho-physically healthy individuals are more likely to engage in social activities, which may positively reflect in SRH [46]. On the other hand, social interaction promotes both physical and mental health, resulting in good SRH [47].

The role of MPCE on SRH was insignificant in rural settings. If we see the backgrounds of rural residents, we can observe that they are more likely homogeneous in terms of expenditures on food and non-food items, irrespective of income level, due to the availability of limited services compared to urban counterparts [48]. Therefore, the result may be insignificant in rural settings. Furthermore, the risk of morbidities is more or similar among rural older individuals due to the similarity in lifestyle and health practices; therefore, the inequality in health status is not substantial based on MPCE status. The results also may be affected by the high rate of generalized reporting of consumption and self-rated health in rural areas. A recent paper titled “Measure for Measure: Comparing Survey-Based Estimates of Income and Consumption for Rural Households” also found the same findings in lower-middle-income countries [49].

In India, the level of poverty, social exclusion, and spatial injustice are more significant among the Muslims, Christians, and lower castes (SCs, STs, and OBCs) which may be negatively affecting their self-rated health [37]. The regional patterns show that older individuals in the south region are more likely to experience poor SRH than their counterparts. In India, the south region is socio-economically forward region where proportion of the older population is higher than in other regions [50]. Therefore, the burden of multi-morbidities (like obesity, stock, etc.) is higher in the southern region, which may positively affect the result [16].

A key strength of this research is that self-rated health is a significant predictor of subjective health among older Indian adults, irrespective of rural and urban settings. Furthermore, marital status and living arrangements are also protective and important mechanisms for determining health status in later life. However, this study has some limitations too. First, the results of this study indicated the self-rated health of older Indian adults and have not stated the regional variations either. Our study only focused on the role of marriage and living arrangements in self-rated health and how it differs in rural and urban settings among older adults in India. But the evidence suggests that comorbidities may lead to poor health conditions in later life [51]. Second, the cross-sectional nature of the data may allow for misreporting of health status. In India, older people rely on their families for financial and physical support in later life. As a result, during the investigation, older persons may be frightened to open up about their health situation in front of their families. A critical subject for future research is exploring the quality of marital status and living arrangements in the moderating role studied in this research in different rural and urban settings. Third, the impact of cognitive decline/ impairments on the association between between marital status/living arrangement can affect the results; therefore a further study is needed to overcome the limitation of the study. Finally, a qualitative study is also needed to understand the mechanism of marital status and care support or healthy lifestyle and its impact on SRH.

## Conclusion

This empirical study contributes to a clear understanding of rural–urban differences in self-rated health with a moderating role of marital status and living arrangements among older adults in India. In terms of marital status and living situation, this study revealed a considerable variation in SRH between rural and urban areas. Furthermore, older individuals with unmarried and living alone status were more likely to report poor health in India, particularly in rural settings. The current research demonstrates the value of promoting the ideas of care and assistance for senior citizens. Older people need specific policy consideration, especially single people living alone. The present study suggests an in-depth investigation is required to explore the mechanism of marital status, living arrangement, and SRH in the Indian context. It was revealed that older people with multi-morbidities and difficulty performing ADLs and IADLs were more likely to have poor SRH. As a result, providing them with top-notch medical care and social support is highly significant. In conclusion, considering their marital status and living arrangements, there is a need to revise the existing social security and health policies for the older population.

## Data Availability

This study was conducted by the MoHFW and the International Institute for Population Sciences (IIPS) in India using a large dataset publicly available on the LASI website (https://www.iipsindia.ac.in/lasi) with ethical standards being followed, including informed consent being obtained by all participants.
